# Can automated treatment plans gain traction in the clinic?

**DOI:** 10.1002/acm2.12674

**Published:** 2019-07-16

**Authors:** Christopher Amaloo, Lane Hayes, Matthew Manning, Han Liu, David Wiant

**Affiliations:** ^1^ Department of Radiation Oncology Cone Health Cancer Center Greensboro NC USA

**Keywords:** automation, eclipse, prostate, RapidPlan, scripting, VMAT

## Abstract

Recently, there has been an increased interest in the feasibility and impact of automation within the field of medical dosimetry. While there have been many commercialized solutions for automatic treatment planning, the use of an application programming interface to achieve complete plan generation for specific treatment sites is a process only recently available for certain commercial vendors. Automatic plan generation for 20 prostate patients was achieved via a stand‐alone automated planning script that accessed a knowledge‐based planning solution. Differences between the auto plans and clinically treated, baseline plans were analyzed and compared. The planning script successfully initialized a treatment plan, accessed the knowledge‐based planning model, optimized the plan, assessed for constraint compliance, and normalized the treatment plan for maximal coverage while meeting constraints. Compared to baseline plans, the auto‐generated plans showed significantly improved rectal sparing with similar coverage for targets and comparable doses to the remaining organs‐at‐risk. Utilization of a script, with its associated time saving and integrated process management, can quickly and automatically generate an acceptable clinical treatment plan for prostate cancer with either improved or similar results compared to a manually created plan.

## 1 INTRODUCTION

Concerning prostate cancer, it has been stated “that all men, if they live long enough, can expect to get the disease.”^1^ Prostate cancer ranks as the second most common cancer for American males behind skin cancer, representing 20% of the cancer diagnoses for men among all specific disease sites^2^ with surgery, radiotherapy, and active monitoring^3^ as typical treatments. Creation of a radiotherapy treatment plan is a complex process that produces a unique result with the potential for disparity in plan quality achieved and planning time invested. Studies have shown plan quality can affect patient outcomes, with a definitive benefit to those techniques that allow dose escalation while limiting high rectal tissue dose to avoid toxicities.^4^, ^5^, ^6^, ^7^

Common external beam treatment modalities utilized for prostate cancer include three‐dimensional conformal radiotherapy (3D‐CRT), fixed gantry angle intensity‐modulated radiotherapy (IMRT), as well as volumetric‐modulated arc therapy (VMAT).^8^ Both IMRT and VMAT are highly conformal, inversely planned approaches that have become the standard of practice for the treatment of prostate cancer.^9^, ^10^ VMAT in particular has experienced a quick and collective rise in part due to increased efficiency and speed of delivery with sustained and pronounced quantitative quality, potentially improved dose homogeneity, and enhanced normal tissue sparing compared to IMRT and 3D‐CRT techniques.^8^, ^11^ Currently, the creation of VMAT plans is still an intricate and time‐consuming process due to complexity of treatment, anatomical deviation, and level of planner expertise. This leads to resultant VMAT plans with large variations in quality metrics and production times.^12^ The net result is an iterative process of attempted dosimetric improvement, potentially incurring excessive time expenditure for minimal clinical return on investment.

One solution designed to reduce planning time while producing consistent, high quality, and clinically acceptable treatment plans is Knowledge‐based planning (KBP).^13^, ^14^ By leveraging dosimetric and geometric information from previous clinical plans, KBP has the capability to reduce the plan variation, and to optimize time investment in a manner typically associated with increased treatment planner expertise.^14^, ^15^ RapidPlan™ (Varian Medical Systems, Palo Alto, CA) is a commercial KBP predictive model that generates estimated dose‐volume histograms (DVHs) for use in photon optimization based on previous patient anatomy and dose distributions for use within the Eclipse treatment planning system (TPS). Recently, Varian released the Eclipse Scripting Application Programming Interface (ESAPI), which allows the end user to gain access to commonly used data elements and actions within Eclipse without any required user interaction. Using the C# programming language, users can then use logical statements and operations to build a framework around generating a complete treatment plan directly from the Aria database.

While field design and inverse planning options can be implemented through ESAPI, each patient has unique anatomical relationships that require intricate digital analysis and complex logic, which causes scripting alone to be a suboptimal approach. Decision‐making in the planning process is highly subjective and remains dependent on the knowledge, experience, and capability of the planner.^16^, ^17^ With the release of Eclipse version 15.5, ESAPI now has the ability to write to the ARIA database. This creates new possibilities to leverage the power automation and KBP simultaneously to move closer to the theoretical ideal plan state without user interaction. Furthermore, stand‐alone access to ESAPI has made it possible for plans to be created, initialized, and optimized outside the context of the current TPS user interface altogether. The purpose of this study is to demonstrate that with this combination of features we can obtain clinically preferred treatment plans for prostate cancer treatment with almost no user intervention.

## 2 MATERIALS AND METHODS

### 2.1 Patient selection, dose prescription, and structure segmentation

A total of 20 prostate patients (received 78 Gy in 40 fractions) treated with VMAT were retrospectively selected for this study. All patients were simulated in the supine position with support and immobilization devices put in place for the lower legs. A computed tomography (CT) scan was obtained with a scan thickness of 2 mm for treatment planning. Patients were scanned from mid‐abdomen to mid‐thigh and imported to the Eclipse TPS for treatment planning. Segmentation was performed to delineate organs‐at‐risk (OARs) and target volumes. OARs defined in the study consisted of the bladder, rectum, and femoral heads as contoured per RTOG 0126 protocol. The clinical target volume (CTV) was delineated as the prostate gland plus the proximal 10 mm of the seminal vesicles. Margins for the planning target volume (PTV) were non‐isotropic, with a posterior expansion of 5 and 8 mm in all other directions. Patients had an average PTV of 143.3 ± 54.5 cc with a range of values between 58.6 and 256.6 cc. Rectal volume average was 69.6 ± 23 cc with a range of 40.7 to 120.1 cc while the bladder volume average was 221.7 ± 166.4 cc, ranging from 74.8 to 830.1 cc.

### 2.2 Treatment planning

Treatment planning goals for the PTV were a minimum of 100% of prescribed dose to 95% of the PTV volume with a maximum dose not to exceed 110%. Planning was performed with Eclipse version 15.5.1 utilizing up‐to‐date versions of the photon optimizer and the anisotropic analytical algorithm (AAA). Original clinical plans (Manual plan, MP) were generated through standard clinical procedure. Fields were aligned upon an isocenter assigned at the time of CT simulation typically near the center of the prostatic space and utilized 2–3 full arcs with planner‐chosen collimator rotations. Additional optimization structures were often applied based upon individual user knowledge to assist in dose shaping, especially in areas associated with PTV and rectal overlap. Approximately, 15–20 optimization dose constraints were created, either individually or through the use of an objective template, with interactive adjustment and modification of limits and weighting as needed. Iterative optimization was employed to help refine dose coverage, target homogeneity, and optimal OAR sparing. Intermediate and final dose calculations were performed at 2.5 mm grid size using heterogeneity correction and AAA. Resultant plans were evaluated based on standardized departmental goals, with a total estimated planning time between 120 and 150 min from initial touch to plan finalization. This time was split between passive tasks such as optimization resolution and dose calculation time, as well as active planner activities such as supplemental contouring, alternate plan creation, active optimizer supervision, and periodic reviews.

A RapidPlan™ model for prostate patients was trained with a set of 80 patients treated with VMAT technique consisting of two full arcs with screening to exclude special circumstances such as nonstandard anatomy or abnormal dose criteria. Outliers were detected using vendor‐provided Model Analytics^TM^ tool. To ensure robustness, each case was analyzed individually to evaluate if removal from the cohort was appropriate. ESAPI‐based scripting was utilized to access the RapidPlan™ model for the automatic plan (AP) generation. The AP script requires a CT data set and radiotherapy structure set objects to exist in the database. When initiated, it checks the integrity of the input data, creates the course, initializes a treatment plan with those basic elements, places the isocenter, enters the prescription according to the electronic records, generates standard two full arc beam arrangement, adds the reference points, and customizes the dose calculation settings. Course, plan, and beam names are automatically rectified from default values to match department‐specific naming conventions.

Once initialized, the RapidPlan™ model is applied to the structure set and DVH estimates are generated. This model automatically generates the optimization parameters based on departmental specifications set by the user during initial model training and that patient's unique anatomy. The optimization process is started, dose calculated, and standard normalization applied. Next, the resulting plan is evaluated against key dosimetric endpoints and if they are not met then additional optimization points are added, and the plan is re‐optimized. The final plan is then saved back to the database and the user is notified of its completion. APs were generated on all 20 test cases without any additional user input, interactive intervention, or post calculation adjustments. The completed plans were verified for minimum target and OAR compliance through a scripted plan checker.

### 2.3 Dose comparisons and clinical review

Plan evaluation was performed by comparing selected dose‐volume parameters between the MP and AP plans. For the PTV, D95 (dose to the 95% of the volume), D98 (dose to the 98% of the volume), the conformity index (CI, ratio of the volume receiving 100% of prescription dose to the PTV volume), homogeneity index (HI, difference of the dose covering 98% of the PTV volume and the dose covering 2% of the PTV volume divided by D_RX_) were selected. The selected parameters were mean dose and V_75Gy_, V_70Gy_, and V_65Gy_ for rectum, and mean dose and V_75Gy_ V_70Gy_, and V_60Gy_ for bladder, respectively.

Statistical analysis was performed to compare the dosimetric differences between AP and MP. Paired Student's *t* test was used to evaluate the statistical significance of all the dose‐volume parameters between the MP and AP. A *P* < 0.05 was considered statistically significant.

To yield a real‐world assessment of the robustness of the AP process, actual patients from a consecutive time‐period were utilized yielding a range of target and normal tissue volumes. The AP script utilized only the PTV and OARs structures without the need for any planning or helper structures.

Treatment plan complexity was evaluated by the use of modulation complexity score (MCS). This algorithm was originally defined to assess the plan complexity and deliverability for step‐and‐shoot IMRT^18^ and was extended to apply to VMAT treatment (utilizing control points of the arc to replace the segments).^19^ MCS calculation and analysis was implemented via a plug‐in script for the Eclipse TPS. Higher values of MCS indicate reduced complexity with increased plan deliverablity.

A group of eight clinicians including medical dosimetrists, medical physicists, and radiation oncologists blind‐reviewed and compared each of the 20 paired test cases to determine clinical suitability as well as general preference for each plan set. Clinicians were tasked to select a preference for a given undisclosed and unidentified plan with the option of no preference. Individual plan comparison techniques varied by reviewer, but plan evaluation was generally achieved through direct examination of isodose lines, coverage for target, location and degree of hot spots, OAR sparing on a DVH comparison, splay of low dose, Monitor Unit (MU) and MCS comparison, and estimated treatment time. A comment section was provided to yield some insight into the review process. This approach was taken to help assess the performance of the algorithm with regard to intricacies beyond meeting general dosimetric guidelines.

## 3 RESULTS

**Figure 1:**
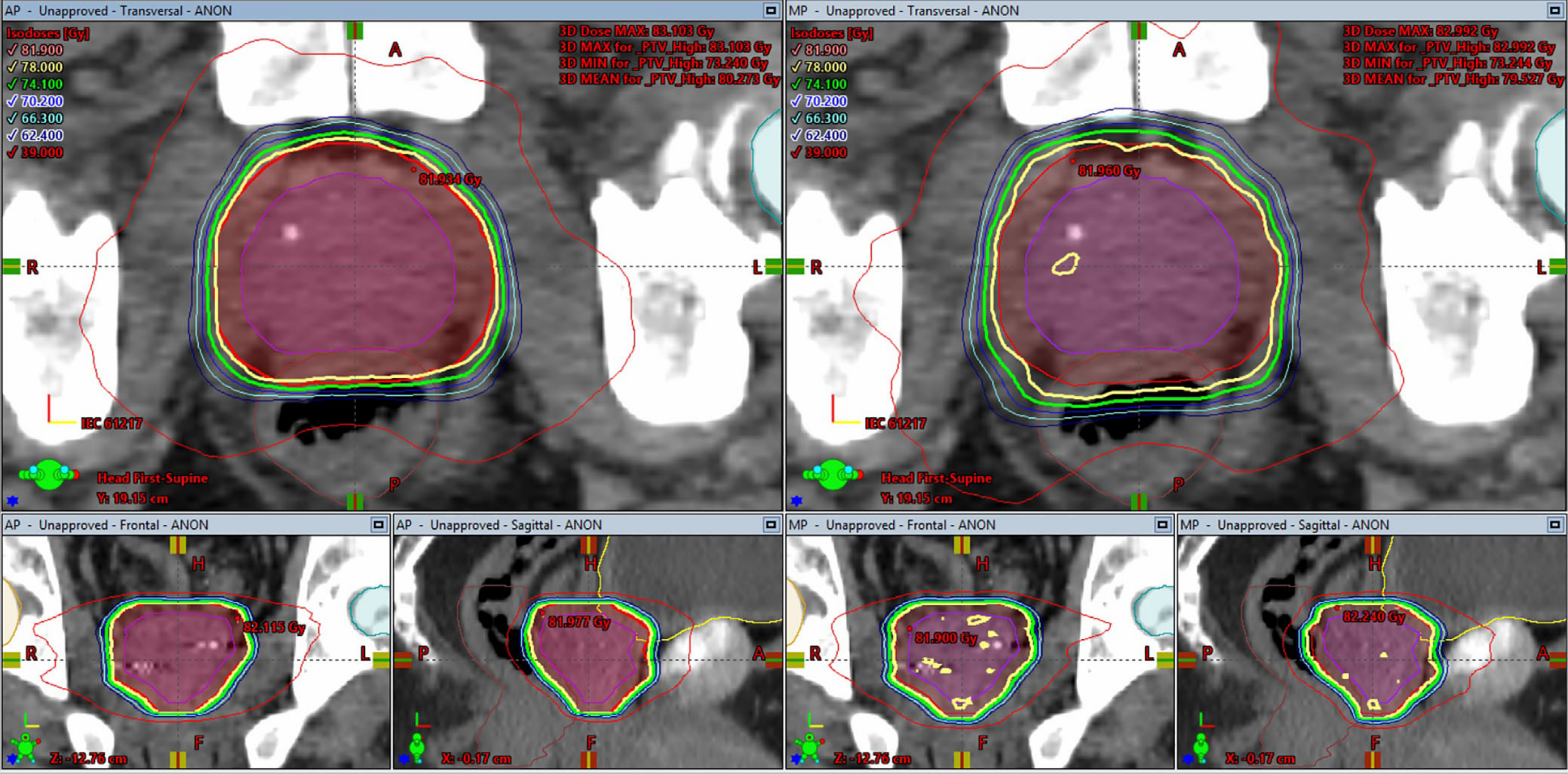
Visual comparison of isodose distributions between the automatic plan (left) and manual plan (right) on the coronal, sagittal, and axial isocentric slices for one representative patient

A representative coronal, sagittal, and axial isodose distributions for the AP and MP are shown in Fig. 1. Qualitative inspection shows that the prescription dose (yellow line) is more conformal for AP compared to MP. The volume 50% of the prescription dose (red line) is comparable between the two plans with slightly more left and right splay for AP but less in the anterior and posterior directions. Location of the intersection of the 50% isodose line and the rectum is closer to the target for AP, indicating greater sparing of the rectal volume. The mean HI was slightly higher for AP plans; however, the target minimum dose shows improvement, easily visualized by the jagged yellow prescription isodose line, which shows “holes” of lower than prescription dose throughout the target volume on the MP.

For all 20 patients, both AP and MP plans meet departmental guidelines for OAR sparing, as well as the minimum target coverage. Table 1 compares average values of selected dose‐volume parameters between AP and MP for PTV, rectum and bladder, as well as *P*‐values from paired Student's *t* test. All percent values are based normalized to prescription dose or total structure where applicable. Maximum dose (D_max_) referred to throughout this work was calculated as the maximum dose to 0.035 cc of a given structure.

**Table 1:** Results from the statistical analysis of the collected data comparing automatic plans to manually generated plans. Significant values from the paired *t* test are highlighted and checks indicate which planning technique showed a more favorable trend

	AP	MP	*P*‐value	AP better	equal	MP better
PTV						
D_95_ (%)	99.99 ± 0.02	99.99 ± 0.02	0.917		✓	
D_98_ (%)	99.75 ± 0.16	99.81 ± 0.22	0.199		✓	
Max (Gy)	82.79 ± 0.53	82.32 ± 0.61	0.003			✓
HI	4.51 ± 0.50	4.23 ± 0.83	0.105		✓	
CI	0.98 ± 0.00	0.98 ± 0.01	0.588		✓	
Rectum						
V_60Gy_ (%)	11.73 ± 5.50	13.57 ± 5.08	9.05E‐05	✓		
V_65Gy_ (%)	9.66 ± 4.72	11.32 ± 4.37	6.37E‐05	✓		
V_70Gy_ (%)	7.59 ± 3.81	9.01 ± 3.62	1.03E‐04	✓		
V_75Gy_ (%)	5.15 ± 2.64	6.39 ± 2.75	0.000	✓		
Mean (Gy)	26.99 ± 5.38	29.00 ± 4.49	0.004	✓		
Max (Gy)	79.75 ± 3.15	80.57 ± 1.62	0.051		✓	
Bladder						
V_60Gy_ (%)	13.22 ± 8.79	13.93 ± 9.44	0.126		✓	
V_70Gy_ (%)	9.72 ± 7.02	10.10 ± 7.31	0.259		✓	
V_75Gy_ (%)	8.01 ± 6.10	8.10 ± 6.07	0.703		✓	
V_80Gy_ (%)	3.91 ± 3.13	2.90 ± 2.53	0.058		✓	
Mean (Gy)	25.31 ± 10.84	25.70 ± 10.73	0.475		✓	
Max (Gy)	82.22 ± 0.64	81.74 ± 0.69	0.009			✓
Plan						
Total MU	719.2 ± 67.1	737.3 ± 84.6	0.427		✓	
MCS	0.41 ± 0.02	0.37 ± 0.05	0.001	✓		

### 3.1 PTV coverage, HI, and CI

**Figure 2:**
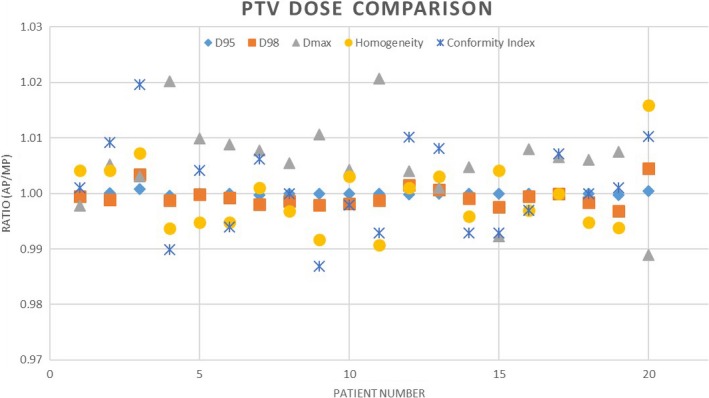
Comparison chart showing the ratio of the planning target volume dose metrics from the automatic plan to the manual plan

The ratio of the AP and MP for D_max_, D_95_, D_98_, HI, and CI for each case is shown in Fig. 2 for the PTV. For D_max_ and HI with values <1 indicating better performance for AP, 1 indicating parity, and greater than 1 indicting better performance of the MP. For D_95_, D_98,_ and CI, this pattern is reversed as an optimal plan maximizing these values.

### 3.2 OAR sparing

**Figure 3:**
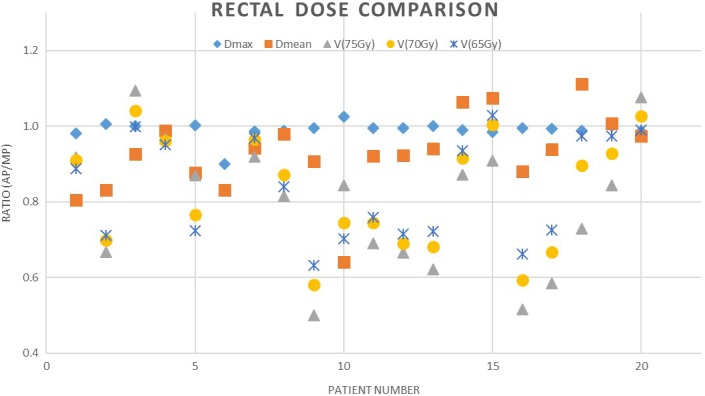
Comparison chart showing the ratio of the rectum dose metrics from the automatic plan to the manual plan

There was significant improved sparing of the rectum for the comparison points V_70Gy_, V_65Gy_, V_60Gy_, and V_55Gy_ (*P*‐values from Table 1). The ratio of the AP and MP for D_max_, D_mean_, V_75Gy_, V_70Gy_, and V_65Gy_ for each case is shown in Fig. 3, again with values less than 1 indicating better performance for AP, 1 indicating equivalence, and greater than 1 indicting better performance of the MP.

**Figure 4:**
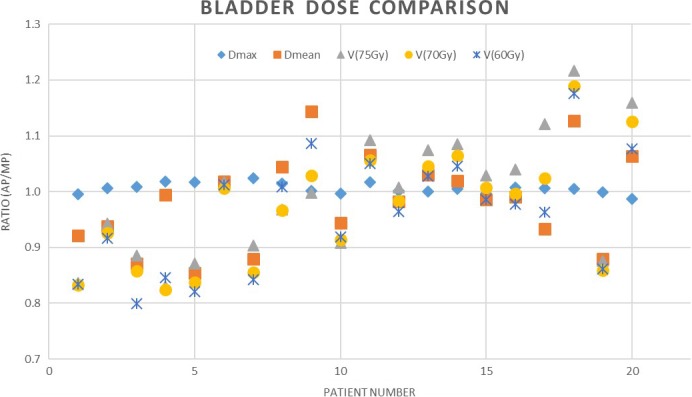
Comparison chart showing the ratio of the bladder dose metrics from the automatic plan to the manual plan

The results for bladder are summarized in Fig. 4, indicating the ratio of the AP and MP for D_max_, D_mean_, V_75Gy_, V_70Gy_, and V_60Gy_. No significant differences are found between AP and MP for all the dose‐volume parameters for bladder in this study with the exception of the max dose (see Table 1).

### 3.3 Planning efficiency, MU, and MCS

The average total MU for the MP was 737.3 ± 84.6 MU while the AP was 719.2 ± 67.1 MU. The plan complexity using MCS was 0.41 ± 0.02 for the AP compared to 0.37 ± 0.05 for the MP. These results show that there was no marked increase in complexity and some statistically significant improvement in terms of the overall Multileaf Collimator (MLC) complexity using the automated approach.

### 3.4 Blind review

**Figure 5:**
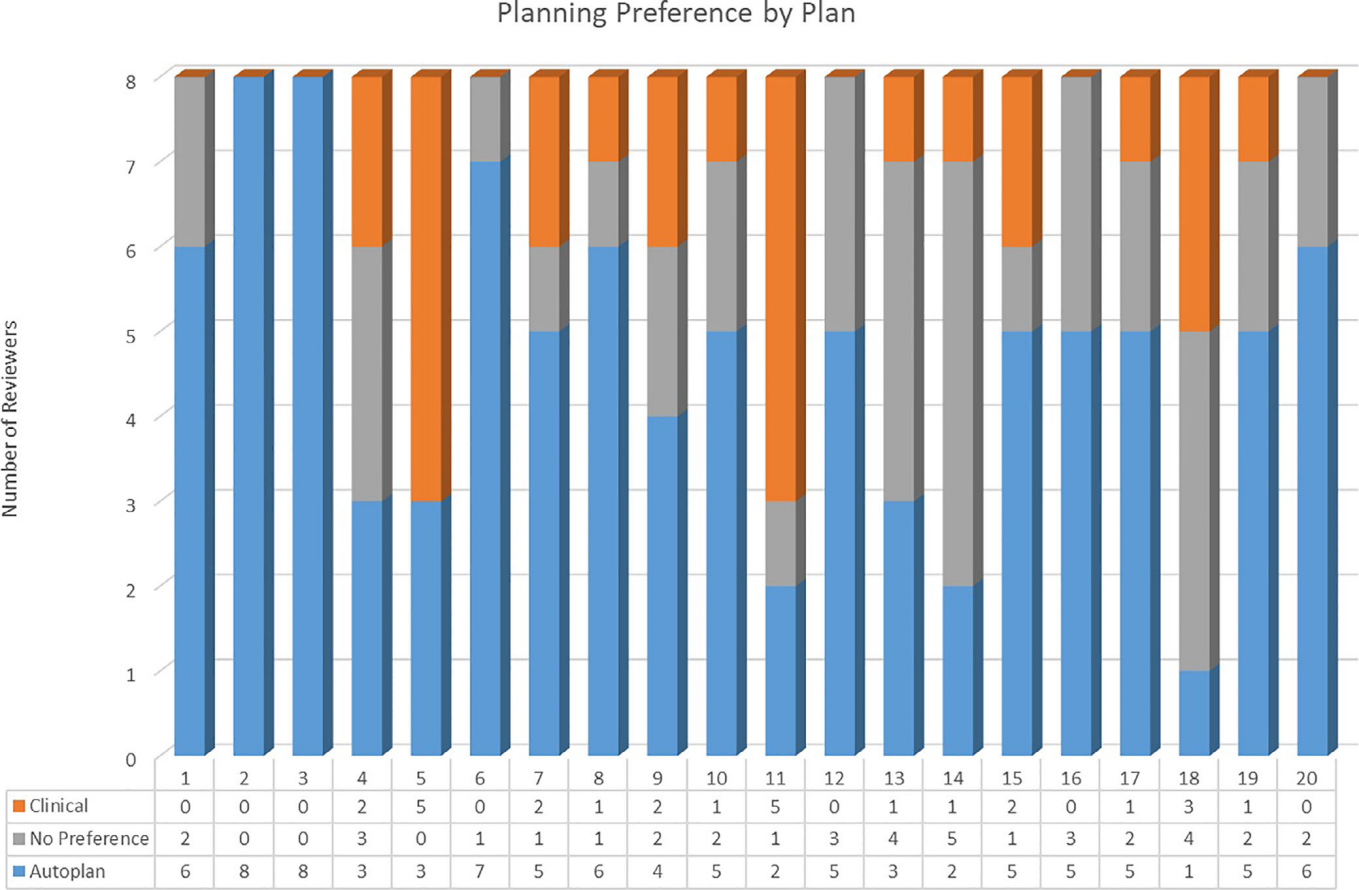
Bar graph showing the preferences between the two plan types by radiation oncology professional on blind review. Reviewers were giving the option to choose plan A, B, or no preference. No preference was interpreted as a vote of confidence for the new technique

The results of the blind review are summarized in Fig. 5. Counting no preference as a vote of confidence for the AP, 90% of the 20 AP test cases received a majority vote. Even excluding these tie votes, 85% of the AP plans received a majority selection as the preferred plan choice. As shown in Fig. 5, there were seven patients where the AP choice either tied or exceeded the MP plan by all reviewers. Conversely, in every case, there was at least one reviewer who viewed the AP treatment as superior to the MP option and there was always a dissenting opinion even when the MP was the majority favorite.

## 4 DISCUSSION

The focus of this study concerns the planning process of radiation therapy with discussion focused on factors that have direct impact on this issue. The primary benefits of AP from this perspective are the consistent improvement in plan quality compared to MP, sizeable time savings, and commensurate reduction in required resources.

All PTV metrics were dosimetrically similar between AP and MP with the exception of max dose to the target. Our clinical experience with intervention‐free inverse planning with Eclipse KBP does produce plans with slightly greater maximum dose. The AP technique accessing the KBP engine required care in managing the maximum dose to a small volume; however, the manual planner ability to improve this number may have been at the overall expense of other parameters. This increase did have statistical significance (*P* = 0.003), nevertheless the absolute dose difference was relatively small. The consistency of values in Fig. 2 hovering near the parity line shows the similarity in PTV coverage between AP and MP approach.

The results of Table 1 show increased sparing at points of interest within the rectal volume. This sparing is statistically significant as shown by the analysis. The superior sparing of the rectum is also visually apparent in Fig. 3 by the majority of points plotted beneath the parity line. D_max_ was similar between AP and MP, but all other factors show a majority of improvement with AP.

Bladder max dose was also improved for the MP compared to AP with statistical significance (*P* = 0.009). Other bladder values were similar between planning method. Bladder data in Fig. 4 show random results, with neither AP nor MP providing consistently improved results. While sparing of all OAR's is important, the relative value of the bladder increase compared to the rectal sparing is regarded as an acceptable compromise.

Overall time savings associated with the script usage were substantial. Total time dedicated to complete a prostate plan was reduced from approximately 120 to 20 min. The active planning tasks were reduced from 60 min to as little as 5 min by utilizing AP over MP. Due to the retrospective nature of the study, exact planning times for the MPs used in this work were able to be not recorded. An estimation based on planner survey and observation was utilized to determine general treatment planning times.

This automated process, consisting of some simple data entry and a singular button click, transforms an arduous manual process to one that is essentially resource‐free. There is clear value delivered by scripting and KBP in the form of greater speed of planning, increased consistency of plan quality, and decreased reliance on planner expertise. Yet, without clinical implementation, these gains are pointless. Implementation of advanced technology in the radiation oncology clinical setting can prove problematic due to factors such as leadership, training standardization, expertise, resources, as well as psychological resistance to change.^20^ Automation provides an opportunity to solve some of these major problems. The solution we have outlined provides a standardization through scripting that promises all patients receive a minimum technical standard and appropriate initial design. The KBP model customizes the plan outcome by pushing toward the optimal plan for a patient's specific anatomy using estimates from the plan library. It has been our experience that this combination, automation and KBP, results in a superior rate of adoption of KBP, enables the fabrication of clinically relevant plans in a fraction of the time, and facilitates the ability of expert staff.

Additionally, this progression not only replicates the process of planning but also provides a complete and AP solution, from origin and initialization through optimization, normalization, and review. This increases the overall value of this process of automation, greatly reducing the time required from simulation to plan review by combination of these two powerful tools.

Even if the AP is not a perfect solution for all patients, it still provides an excellent starting point for further manual optimization and plan improvement. The processes of setting the isocenter, creation of fields with following appropriate naming conventions, and design of optimization goals all being performed without user input has intrinsic value to busy, high‐performing departments. Additionally, the application of KBP constraints, initial normalization, and creation of some optimization structures through the AP script such as a PTV expansion to help with coverage can assist if further optimization is necessary. It should prove to be a simple process to verify optimization weighting, ease or increase constraints, and manage priorities to help achieve a more idealized isodose distribution.

The results of the blinded review were extremely compelling. Given the overwhelming vote of confidence in the AP‐produced outcome, there is little reason not to utilize this solution as a primary approach. This revelation has proven to be a key motivating factor for planners in our clinic to adopt KBP solution through AP for clinical use where manual implementation alone was not.

Radiation treatment planning for prostate cancer is a common, well‐established treatment site with clear clinical guidelines and was therefore ideal for a proof of concept study into automation‐ and knowledge‐based planning. While these plans may be seen as straightforward to experienced planners, the broad clinical application of KBP suggests that application of this technique may easily extend to other more challenging anatomical sites. The ability of the system to overcome complexities, which were formerly insurmountable boundaries for a solely computerized system, shows promise for further exploration.

## 5 CONCLUSION

Treatment planning scripts can be a valuable tool in the creation of plans based on both improved efficacy and efficiency of treatment. Automated plans using KBP were able to produce plans of clinical quality meeting OAR constraints and target coverage requirements for a random cohort of prostate cancer patients. Target coverage for AP was equivalent to MP while also significantly improving sparing of the rectum at no cost to other established metrics. Utilizing an automated planning approach reduced active planning time to under 5 min and total planning time to 20 min including optimization and calculation time. Utilization of the ESAPI allows an automated approach to complete treatment plan production while simultaneously driving the implementation of a KBP, which is linked to increase quality. Freed resources become available for allocation toward other planning or professional duties, further increasing the efficacy and efficiency of treatments produced, while maximizing the skills of individual treatment planners. Finally, on blind review for 18 of 20 cases, the AP solution produced results that met or exceeded the MP technique for a majority of reviewers suggesting this should be the preferred process for initiation of treatment plans. Although this study was limited to prostate cancer treatment as a proof of concept, we expect that the design is extensible to other anatomical sites and we plan to explore that in future work. Neither resource alone, scripting, or KBP have proven to be a complete or independent solution for automated plan generation but together we believe they can become an invaluable clinical tool in today's demanding healthcare environment.

## CONFLICTS OF INTEREST

The authors do not have any conflicts of interest to report.
